# Physicians Wanted in Norfolk

**Published:** 1863-10

**Authors:** W. H. Brooks

**Affiliations:** Mayor of Norfolk, Va.


PHYSICIANS WANTED IN NORFOLK.—The following, taken from the *Norfolk Virginian*, shows a demand for physicians in the South:

"We are requested, by the Mayor of Norfolk, to say that several physicians are wanted in this city, and can obtain immediately a good practice. The old physicians being required by an act of the Legislature of Virginia to take the oath of allegiance to the United States Government, refuse to do so generally, and consequently there is but one doctor in the city qualified to practice.

Loyalty and regular diploma are the qualifications required. Apply to

W. H. BROOKS, Mayor of Norfolk, Va."

☞ Medical men who wish to join the State Corps of Volunteer Surgeons for service during the exigencies of a battle, should make application to the Surgeon-General of the State in which they are residing.

There are a number of Surgeons and Assistant Surgeons required for the regiments of U. S. Colored Troops. The pay is the same as that of other regimental medical officers, Surgeons, $163.00, and Assistant Surgeons, $112.83 per month. They must be examined by a Medical Board previous to appointment, and the general principles of examination are the same as those observed in the examination of Assistant Surgeons of Volunteers. Application should be made to the Surgeon-General, U. S. A., at Washington, D. C., for permission to come before the Board.

